# Kinetics and perception of basketball landing in various heights and footwear cushioning

**DOI:** 10.1371/journal.pone.0201758

**Published:** 2018-08-09

**Authors:** Qiang Wei, Zhao Wang, Jeonghyun Woo, Jacobus Liebenberg, Sang-Kyoon Park, Jiseon Ryu, Wing-Kai Lam

**Affiliations:** 1 Department of Physical Education, Tangshan Normal University, Tangshan, China; 2 Qing Gong College, North China University of Science and Technology, Tangshan, China; 3 Motion Innovation Centre, Korea National Sport University, Seoul, Korea; 4 Institute of General Kinesiology and Athletic Training, University of Leipzig, Leipzig, Germany; 5 Department of Kinesiology, Shenyang Sports Institute, Shenyang, China; 6 Li Ning Sports Science Research Center, Li Ning (China) Sports Goods Co. Ltd, Beijing, China; Nanyang Technological University, SINGAPORE

## Abstract

**Background:**

The previous studies on basketball landing have not shown a systematic agreement between landing impacts and midsole densities. One plausible reason is that the midsole densities alone used to represent the cushioning capability of a shoe seems over simplified. The aim of this study is to examine the effects of different landing heights and shoes of different cushioning performance on tibial shock, impact loading and knee kinematics of basketball players.

**Methods:**

Nineteen university team basketball players performed drop landings from different height conditions (0.45m vs. 0.61m) as well as with different shoe cushioning properties (regular, better vs. best-cushioned). For each condition, tibial acceleration, vertical ground reaction force and knee kinematics were measured with a tri-axial accelerometer, force plate and motion capture system, respectively. Heel comfort perception was indicated on the 150-mm Visual Analogue Scale. A 2 (height) x 3 (footwear) ANOVA with repeated measures was performed to determine the effects of different landing heights and shoe cushioning on the measured parameters.

**Results:**

We did not find significant interactions between landing height and shoe conditions on tibial shock, impact peak, mean loading rate, maximum knee flexion angle and total ankle range of motion. However, greater tibial shock, impact peak, mean loading rates and total ankle range of motion were determined at a higher landing height (*P* < 0.01). Regular-cushioned shoes demonstrated significantly greater tibial shock and mean loading rate compared with better- and best-cushioned shoes (*P* < 0.05). The correlation analysis indicated that the heel comfort perception was fairly associated with impact peak and mean loading rate regardless of heights (*P* < 0.05), but not associated with tibial shock.

**Conclusions:**

Determination of shoe cushioning performance, regardless of shoe midsole materials and constructions, would be capable in order to identify optimal shoe models for better protection against tibial stress fracture. Subjective comfort rating could estimate the level of impact loading in non-laboratory based situations.

## Introduction

Basketball is a popular sport with more than 450 million players worldwide [1]. Jump landing is an indispensable movement during basketball games. On average, each player performs 70 jumps in a game [2] and experiences impact up to nine times body weight during the landing phase of each jump [2]. Failure to attenuate repetitive impacts would inevitably lead to excessive loads on the lower extremities [3–4]. Therefore, studying landing impact characteristics would help to predict lower extremity injuries in basketball [5].

Tibial stress fracture (TSF) is one of the common overuse injuries in basketball players, as it accounts for 10 injures in every 1000 games [3, 6]. Even though the etiology of TSF has been extensively investigated, the underlying mechanism has not been well determined. Previous research suggests that TSF may relate to higher levels of tibial shock [7], impact peak [8] and loading rate [8] in running population. During a game, basketball players execute various jump activities for layup, shot-blocking and shooting [9, 10], which may cause different intensities of impact loading (e.g. related to height and upper movements) across landing movement [10]. In basketball literature, these risk factors are influenced by landing height [9] and footwear properties (e,g, midsole densities) [10, 11]. In brief, lower landing heights and softer midsoles would reduce the loading on the lower extremities.

Appropriate sport footwear should effectively attenuate impact forces to lower risks of overuse injuries in various impact activities [12–13]. While midsole densities have been extensively investigated in landing activities, some studies have showed that the impact load experienced by participants did not change systematically across alterations in footwear cushioning performance [10, 11, 14]. One study found higher landing impacts in harder midsole shoes (Shore C70) compared with softer midsole shoes (Shore C40) [11]; In contrast, other studies revealed the similar landing impacts between softer midsole shoes (Shore C38, Shore C45) and harder midsole shoes (Shore C57, Shore C65) [10, 14]. One possible explanation of these contrasting findings is due to the differences in how landing movements are conducted in the studies as well as the range of midsole densities used. Another explanation is that the midsole densities alone to denote the cushioning capability of a shoe seems to be over simplified. In general, the cushioning properties should be incorporated with material viscoelasticity, midsole thickness and structure among shoe models. While cushioning performance is rated as the most important footwear feature in basketball players [15], investigating shoe cushioning effect on landing are limited and remain inconclusive. Therefore, comparing shoe mechanical cushioning performance, rather than quantifying only midsole hardness, would allow comparison across studies which used different shoe models.

Previous studies reported the differences in landing heights and/or types might have contributed to the distinct biomechanical and perceptual responses of different cushioning performances [10]. Hence, this study sought to compare tibial shock, impact peak, vertical loading rate, knee and ankle angles and comfort perception of basketball players landing from different heights and with basketball shoes of different cushioning performances. Since shoe cushioning is an important regulator of footwear comfort [16] and suggests to have good correlation with kinetics variables [17], this study also sought to examine the correlation of comfort perception with the kinetics variables. Based on the previous findings, it is hypothesized that lower impact loading would result from lower landing heights or a pair of better cushioned basketball shoes. It is also expected that comfort perception would be associated with vertical GRF, loading rate and tibial shock.

## Materials and methods

### Participants

Nineteen male basketball players [mean age 25.0 (2.3) years; height 1.8 (0.05) m; mass 74.4 (6.5) kg] were recruited for this study. All participants had at least four years of competitive basketball experience and attended practice for more than four hours per week. They had no lower extremity injury during the past six months prior to the start of the study. Ethical approval was granted by Li Ning institutional review committee (IRB-2015BM005). All participants signed an informed consent form prior to the start of the study.

### Footwear conditions

Three pairs of basketball shoes were selected based on their respective cushioning performance according to the standard mechanical impact attenuation test (ASTM protocol F1976-13). In brief, thirty mechanical impact trials were performed at the center of the heel region with an 8.5-kg mass dropping from a 50-mm height. The trials from 25 to 30 were averaged for the calculation of mechanical impact scores of each shoe condition. This standard assessment procedure allowed objective judgment of shoe cushioning for the cross-studies comparison [10, 18]. The test shoes were classified as best-cushioned shoe (9.8 g-force), better-cushioned shoe (11.3 g-force), and regular-cushioned shoe (12.9 g-force), based on the currently available basketball shoes. Shoes with lower impact score indicate better shoe cushioning.

### Procedure

After completing the anthropometric measurements, players wore a new pair of standard socks and performed a 10-min warm-up, which included stretching and jogging with their own basketball shoes. A tri-axial accelerometer (DTS 3D, Noraxon, AZ, USA) was securely fixed on the antero-medial aspect of the distal tibia, as described by previous studies [9, 19, 20]. In brief, the location was approximately 15 cm in the superior direction from the medial malleolus where the first even/flat surface was present. The axial axis of the accelerometer was aligned along the longitudinal axis of the tibia. Double-sided adhesive tape with an elastic bandage was applied by the same investigator to securely fix the accelerometer in order to minimize subcutaneous tissue movements, assuring that the application of the bandage did not alter the location of the accelerometer. In addition, fourteen reflective markers (diameter 14 mm) were then placed over the right thigh, leg and shoe according to the previous studies [21].

After a standardized warm-up protocol, participants were asked to perform five drop landing trials from different height conditions (0.45 m vs. 0.61 m) with different footwear conditions (regular, better vs. best-cushioned). In brief, participants were instructed to remain erect and look forward while positioning their arms across their chest in order to reduce postural sway [10]. Landing movement was initiated by taking a small step forward with the right leg off the platform and then landing with their right leg on the right force plate and left leg on the adjacent force plate [9–11]. A trial with a toe-to-heel landing pattern with good balance and clean footfalls on the force plate (AMTI, Watertown, MA, USA) was regarded as a successful trial. All test conditions were randomized using an online programme (www.random.org). They were allowed to have three familiarization trials prior to data collection. Two minutes of rest were given between each condition to allow the participants to rate their perception of heel comfort on a 150-mm Visual Analogue Scale (VAS), with not comfortable at all (0 mm) to most comfortable (150 mm) [10, 22].

### Data analysis

**Fig 1:**
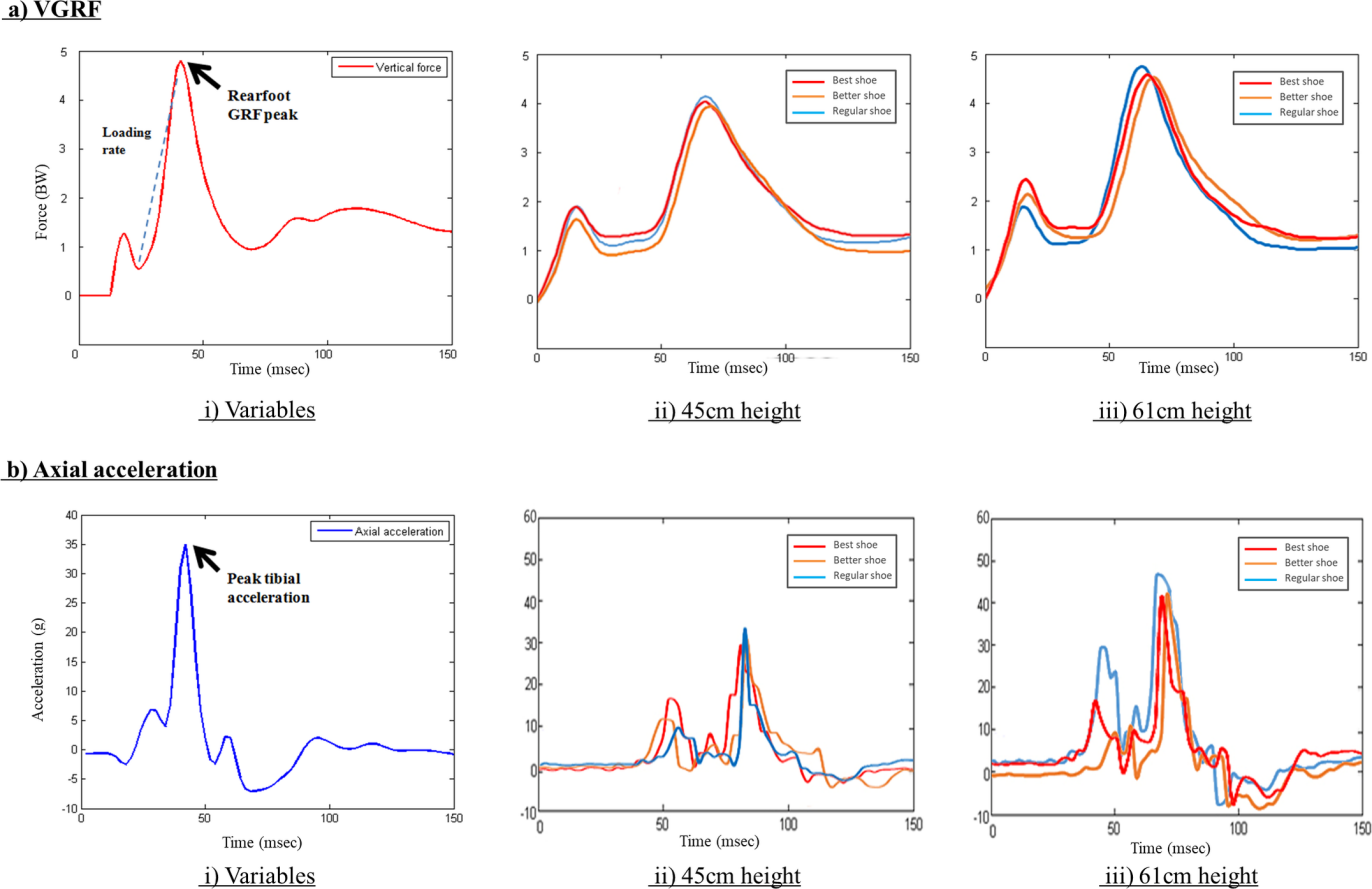
Representative vertical ground reaction force (VGRF) and axial acceleration versus time profiles of a drop landing in different shoe and height conditions.

Tibial acceleration and vertical ground reaction force (GRF) were recorded at 1,500 Hz and 1,600 Hz, respectively (Fig 1). Motion data were captured using an 8-camera system (Vicon, Oxford Metrics, Oxford, UK) at 240 Hz. To synchronize all tibial acceleration, GRF and motion trajectory signals, participants were asked to strike the force platform hard with their right foot before data acquisition of each trial. A spline interpolation was performed for minor missing marker trajectories using three frames before and after the missing data. A customized MATLAB (Mathworks, Inc., Natwick, MA, USA) code was used to process all kinetic and kinematic data. Kinetics data were filtered with a fourth-order Butterworth low-pass filter at 50 Hz [10] and vertical ground reaction force data were normalised to body mass.

The onset of the impact phase was determined when the vertical GRF first exceeded a 10N threshold. Mean loading rates were calculated from 0% to 100% before the second peak [10, 23]. Tibial shock was defined as the maximum positive axial acceleration after foot contact in all landing trials. Knee and ankle joint angles were defined as the orientation of one distal segment (i.e., shank) relative to the proximal segment (i.e., thigh). Only maximum knee flexion angle and total ankle range of motion were evaluated in the present study as it is the primary variable of interest during landing activities [9].

All statistical analyses were performed using SPSS programme (SPSS, Version 20.0, IL, Chicago). Being robust to moderate violations of normality observed in most of the data, a 2 (landing height) x 3 (cushioning) ANOVA with repeated measures was used for statistical analysis of tibial shock, peak and mean loading rate of GRF, maximum knee angle and total ankle range of motion. Another one-way ANOVA with repeated measures was used to determine if there is significant difference in shoe comfort perception. Greenhouse-Geisser’s epsilon adjustment was used in all cases when Mauchley’s test indicated that the sphericity assumption had been violated. Bonferroni corrected post-hoc tests were performed to correct multiple measurements, with the level of significance at *P* = 0.05 for all cases. In addition, Pearson products correlation test was performed to examine if shoe comfort perceptions were correlated with kinetics variables, while pooling all data from all shoe conditions. The correlation (*r*) was classified as little/no (> 0.0 to ≤ 0.25), fair (> 0.25 to ≤ 0.50), moderate to good (> 0.50 to ≤ 0.75) and good to excellent (> 0.80 to ≤ 1.00) relationship [24].

## Results

There were no significant interaction effects between height and shoe on tibial shock, impact peak, mean loading rate, maximum knee flexion angle and total ankle range of motion (Table 1). However, greater tibial shock, impact peak and mean loading rate, and larger knee flexion angle and total ankle range of motion were found at a higher landing height (*P* < 0.01). Significant shoe effects were found in tibial shock (*P* < 0.01), mean loading rate (*P* = 0.02) and total ankle range of motion (*P* < 0.01). In the post-hoc analysis, we found that participants wearing regular-cushioned shoes experienced significant greater tibial shock and mean loading rate compared with better and best-cushioned shoes. Participants wearing better-cushioned shoes had demonstrated significantly larger total ankle range of motion than the other shoe conditions (*P* < 0.01).

**Table 1:** Landing kinematic, GRF and tibial acceleration variables during each landing height expressed in mean (standard deviation). *n*^2^ = partial eta squared; *β* = observed power (Table A in S1 File).

		Shoe			Interaction			Landing height			Shoe			
Variable	Height	Best (A)	Better (B)	Regular (C)	*P*	*η*^2^	*Β*	*P*	*η*^2^	*β*	*P*	*η*^2^	*β*	*Post* *Hoc*
Tibial shock (g)	L	27.32 (9.50)	29.37 (7.64)	32.34 (8.81)	.99	.00	.05	**< .01**	.83	1.00	**< .01**	.43	.85	C>A
	H	40.93 (13.30)	43.34 (12.43)	46.17 (13.37)										
Impact peak (BW)	L	4.02 (0.86)	3.96 (0.88)	4.21 (0.77)	.60	.02	.11	**< .01**	.85	1.00	.17	.09	.36	
	H	4.58 (0.68)	4.50 (0.85)	4.69 (0.62)										
Mean loading rate (BW/s)	L	136.79 (47.83)	138.67 (55.21)	163.97 (54.61)	.61	.03	.13	**< .01**	.69	1.00	**.02**	.21	.75	C>A
	H	179.04 (47.74)	188.26 (70.44)	207.01 (63.47)										
Maximum knee angle (^o^)	L	63.40 (19.54)	63.87 (19.89)	63.94 (19.18)	.16	.10	.37	**< .01**	.79	1.00	.35	.02	.10	
	H	74.77 (24.63)	71.61 (23.13)	72.63 (21.76)										
Total ankle range of motion (^o^)	L	44.62 (7.08)	54.50 (6.86)	44.96 (7.19)	.54	.03	.12	**< .01**	.43	.93	**< .01**	.79	1.0	B>A B>C
	H	46.18 (6.21)	55.17 (6.54)	45.98 (7.26)										
Heel comfort perception		10.19 (2.45)	10.12 (3.14)	10.31 (2.74)							.96	.00	.06	

(L = low (45cm); H = high (61cm); Significant *P*-values (*P* < .05) are shown in bold)

For heel comfort perception, there was no significant difference between the shoe conditions (Table 1). For the correlation analysis, the comfort perception was associated with impact peak and mean loading rate regardless of heights (*P* < 0.05), but not associated with tibial shock in any of the height conditions (Table 2). According to the relationship suggested by the previous study [24], only a fair relationship was determined between cushioning perception and ground reaction force variables.

**Table 2:** Pearson correlation (*r*) and *p*-values between cushioning perception and other test variables (Table B in S1 File). Significant *P*-values (*P* < .05) are shown in bolded.

		Landing height	*P-*value	*r*	Relationship level
Comfort perception vs Tibial shock Comfort perception vs Impact peak Comfort perception vs Mean loading rate		45cm 61cm 45cm 61cm 45cm 61cm	.160 .120 **.046** **< .010** **< .001** **< .001**	.33 .21 .27 .37 .44 .49	Fair Little/no Fair Fair Fair Fair

## Discussion

The present study investigated the landing biomechanics in basketball players at different height and footwear conditions. As we expected, basketball players experienced greater impact loading in terms of tibial shock, impact peak, and mean loading rates, and larger knee and ankle flexion angles at higher landing heights. These findings are in accordance with previous basketball shoe studies [9, 25]. It could be explained by the increased knee flexion angles and/or muscular activation when landing from higher position [9, 25, 26]. Devita and Skelly (1992) had participants performing ten successful trials of soft (larger knee flexion angle) and stiff landing techniques (smaller knee flexion angle) at the same landing height. They found the participants with soft landing techniques associated with lower vertical impact forces, suggesting that impact attenuation in landing would be regulated and influenced by knee kinematics.

Tibial stress fracture is one of the most common overuse injuries in basketball players [3, 6]. Shock absorption and impact attenuation are the primary considerations in sport footwear design to lower the risks of impact-related injuries [12, 15]. In this study, the relationship between shoe cushioning performance and impact loading was generally supported with our original hypothesis. It supports that superior cushioned shoes would result in lower impact loading and tibial shock on lower extremities compared with the inferior cushioned shoes. This implies that studying shoe mechanical cushioning performance, rather than quantifying only midsole hardness [9–11], would allow comparison across studies between different shoe models with a variety of materials and structures. This can provide common baselines for sports trainers, athletes and footwear developers to evaluate footwear performance.

Interestingly, the present results showed the fair correlation between comfort rating scores and impact loading (in both landing heights), which provides partial support on the previous studies on shoe cushioning [13]. However, participants wearing better-cushioned shoes experienced lower impact loading, lower tibial shock and larger ankle range of motion, but at the same time participants did not differentiate comfort perception amongst the shoes. One plausible explanation is that the human body might unconsciously promote their movement patterns or motor program to avoid high impacts in different landing activities. For instance, habitual rearfoot strike runners would change their natural movement pattern to midfoot/forefoot striking pattern when running in barefoot condition without prior instructions [27]. In a similar vein, when landing from a higher landing position, basketball players would unconsciously regulate their knee joint kinematics to avoid high impacts [11]. It would be beneficial to study constraints on the knee joint kinematics with simple instructions to establish the relationship among shoe cushioning, landing kinetics and comfort perception [28]. The alternative explanation would be that the somatosensory system of the skin was generally lower across the foot regions [29]. To confirm this speculation, future studies should examine the relationship between the thresholds of impact forces and perception rating in various groups of participants (e.g., expertise, sport types and body mass).

When interpreting our results, it is important to consider a few limitations in our study. Firstly, we included only male recreational basketball players and did not consider their playing positions [15]. Hence, our findings cannot be generalized to females, playing level and position of the players. Secondly, we used the typical drop landing protocol to standardize movements across conditions and participants. While this is not typically executed in basketball games, comparison between the present results and other landing movements in basketball should therefore be made with caution. Thirdly, the basketball shoe models used in this study were available in the market, which had different shoe constructions. Considering that cushioning performance incorporates different elements like material viscoelasticity, midsole thickness and structures, studying shoe cushioning would allow for comparing results across studies, especially with different footwear models used among different studies. For this reason, future studies are warranted to investigate the impact loading for other isolated footwear structures (e.g., landing surface area and midsole hardness) as well as basketball related movements.

## Conclusions

Basketball players experience greater impact loading and tibial shock at higher landing height or with inferior cushioned shoe condition. Determination of shoe cushioning performance, regardless of shoe midsole material and construction, may be able to identify the optimal shoe model for better protection against tibial stress fracture. The comfort perception was found to be fairly correlated with impact loading, but not tibial shock, implying that subjective perception rating could be obtained to estimate the level of impact loading in non-laboratory based situations.

## Supporting information

S1 File Table A and Table B provide supporting information for Table 1 and Table 2, respectively.
(XLS)
